# Effects of co-occurrence and intra- and interspecific interactions between *Drosophila suzukii* and *Zaprionus indianus*

**DOI:** 10.1371/journal.pone.0281806

**Published:** 2023-03-30

**Authors:** Larine de Paiva Mendonça, Khalid Haddi, Wesley Augusto Conde Godoy

**Affiliations:** 1 Departamento de Entomologia, Universidade de São Paulo, ESALQ, Piracicaba, São Paulo, Brazil; 2 Departamento de Entomologia, Universidade Federal de Lavras, Lavras, Minas Gerais, Brazil; Inha University, REPUBLIC OF KOREA

## Abstract

In drosophilids, competition and coexistence can impact survivorship, growth, and reproductive output. Here, we evaluated direct competition between two co-occurring fruit flies, the spotted-wing drosophila *Drosophila suzukii* and the African fig fly *Zaprionus indianus*, comparing results from field collections with laboratory experiments. Field collections were conducted to evaluate co-occurrence between species. In the laboratory, different densities of eggs of each species were provided an artificial diet, and intra- and interspecific densities were evaluated regarding biological traits such as development and fecundity. Field collections showed a prevalence of *Z*. *indianus*, followed by other drosophilid species, including *D*. *suzukii*. Pupal survival and adult emergence were higher in *D*. *suzukii* than in *Z*. *indianus* at both intra- and interspecific densities, with decreasing values in response to increased densities. Fecundity did not differ significantly for either species at different intraspecific densities, but when reared together at different densities, *Z*. *indianus* was significantly more fecund than *D*. *suzukii*. Development time showed no significant difference at intraspecific densities, but when reared together, *Z*. *indianus* had longer development times than *D*. *suzukii*. Leslie Matrix projections indicated that *D*. *suzukii* showed practically the same dynamics at intraspecific and interspecific densities, with increasing oscillations at low and intermediate densities and decreasing oscillations at high densities. *Zaprionus indianus* showed a similar oscillation to *D*. *suzukii*, except at intermediate intraspecific densities, when the pattern was cyclic. Low interspecific densities resulted in decreasing oscillations. In the two-choice oviposition bioassays, *D*. *suzukii* females showed no significant preference for diets previously infested or not with either conspecific or heterospecific eggs at different densities. Understanding competitive interactions between co-occurring heterospecific species should be considered when establishing management tactics for spotted-wing drosophila.

## Introduction

Two drosophilid species that damage small fruit have invaded the Neotropics in recent decades. The first invader, the African fig fly *Zaprionus indianus* Gupta (Diptera: Drosophilidae), an exotic drosophilid originating from sub-Saharan Africa [1] that has extended its range from tropical to temperate areas, and showing excellent plasticity to survive in environments with adverse conditions [2]. This fly was reported in Brazil in 1999 in the state of São Paulo [3] and has since spread throughout the country [4–6]. The second invasive drosophilid to arrive in Brazil was the spotted-wing drosophila *Drosophila suzukii* Matsumura, in 2012–2013 [7]. This species is of Asian origin but gained notoriety as an invasive pest of soft fruits with its spread around the world [8, 9]; it was first identified outside Asia in the United States and Europe around the year 2008 [10, 11]. Although reported to occur jointly, these two drosophilids present distinct biological and behavioral traits as well as seasonal phenotypic plasticity that influence their abilities to invade new areas and allow adaptations to different environments under a wide range of temperatures [12]. Indeed, females of *Z*. *indianus* produce around 70 eggs, can pause ovarian development during cold periods without loss of fertility [2, 13], and complete 12 to 16 generations per year. Dissimilarly, *Drosophila suzukii* females are highly fertile and lay more than 200 eggs during their lifetime [14]. They exhibit widely varying longevity (i.e., 35 days at 10°C and 2 days at 30°C), complete 3 to 10 generations per year, and are able to maintain relatively constant fecundity and longevity under low temperatures when afforded a short period of warm temperatures [15, 16]. Furthermore, *D*. *suzukii* has a high dispersal capacity, and depending on microclimatic factors can travel up to 100 m per day [17, 18].

In multiple invasions, the first invading species may gain a clear benefit in terms of abundance compared to subsequent invaders, as it will have more time to adapt to existing resources and challenges in the new land [19]. However, any advantage of the first colonizers will depend on their biological characteristics and their ability to use a wide range of resources, as well as the competitive stress from later-arriving species [20]. In this context, the interplay between the co-occurring *D*. *suzukii* and *Z*. *indianus* merits analysis because of their different biological traits and life strategies [21]. This interplay raises the question of which is the better invasion strategy. To colonize environments with damaged fruits first or to infest undamaged fruits first?

*Drosophila suzukii* is a polyphagous pest attacking a wide range of soft and thin-skinned fruits, which may have facilitated its establishment in different regions [22–24]. Differently from other drosophilids, *D*. *suzukii* damages the surface of fruits with its modified serrated ovipositor [9, 22]. This serrated ovipositor is believed to give *D*. *suzukii* an advantage over other drosophilids such as *Z*. *indianus*, as it allows the spotted-wing drosophila to use healthy fruits that were not previously used by heterospecific competitors [10, 19]. In contrast, *Z*. *indianus* is a secondary pest, able to infest only already-damaged fruits [1]. Despite its difficulty in ovipositing on healthy fruits, the importance of this pest increases when it occurs together with *D*. *suzukii*, since it can use the oviposition sites of *D*. *suzukii* as a gateway for its offspring, overcoming the previous advantage of the latter [25].

Independently of which species is the initial colonizer and whether it colonizes unhealthy or healthy fruits first, both invasive species must initially adjust to the positive or negative effects of low densities upon arrival and possible adverse conditions in the new location before attaining high densities [26]. For invasive species and since biological invasions start with low densities, low densities may be much more beneficial than high densities for establishment [27, 28]. Existing data for fruit flies are not conclusive regarding the best invasion strategy (damaged or undamaged fruit first), but the species’ competitive abilities may indicate the potential of each to persist and prosper under intra- or interspecific competition and to compare the advantages and disadvantages of arriving and consuming unhealthy fruit first or piercing healthy fruit first [29].

Several studies are recognized as classical investigations analyzing and discussing results emerging from interspecific competition in different insect taxonomic groups, such as *Callosobruchus* beetles, *Drosophila* fruit flies, and *Tribolium* beetles. These studies have highlighted that the essential driver of competition is interspecific competition for resources [30]. Competition for food in insects can be triggered mainly when density-dependent mechanisms act on the population [31]. In fruit flies, these mechanisms are observed mainly in ephemeral food substrates, principally fruits, creating the conditions for intra- and interspecific competition [32].

Invader species generally exhibit biological attributes capable of facilitating the colonization process and establishment in new areas, principally the demographic parameters of fecundity and survival and the ability to disperse and adapt to the new conditions imposed by the new environments [33]. Different geographical regions may have seasonal changes capable of altering insects’ behavior in different ways [34]. Local characteristics in the newly invaded environments such as abiotic environmental conditions, including the availability and heterogeneity of suitable habitats, and the implementation of plantations or orchards, resulting in interactions with other native and co-occurring alien species, can determine the success or failure of a species over time [35]. Thus, invader species must adapt to the new environmental conditions when arriving in new areas since humidity, rainfall, photoperiod, wind, and most importantly, temperature will undoubtedly significantly influence the new insects’ distribution, abundance, and behavior [34, 36]. Besides abiotic effects, the biotic effects of the newly invaded area, such as the presence of native natural enemies that can prevent and control invasive species that arrive in low densities, can be challenging for the establishment [37]. Suitable habitat is fundamental for establishing exotic species, which often reach more habitats after invading the first one [38].

The effect of intra- and interspecific competition on the pest *D*. *suzukii* has been evaluated in different studies [39–41]. Intraspecific competition is expected in the field since females of *D*. *suzukii* oviposit more than one egg in the same substrate [9], and the competition has been shown to affect their pupation [39]. Although it is known as a primary pest, the wound left on the fruit by *D*. *suzukii* can attract a new range of pests, exposing the fruit fly to the possibility of interspecific competition [21]. Studies of interspecific competition between *D*. *suzukii* and different species have shown different impacts [41–43], which raises the possibility that the effect of interspecific interaction is dependent on the species that co-occur with *D*. *suzukii*. Studies of interspecific competition between drosophilids may show that the same species does not always have a competitive advantage. In fact, a study investigating the competitive interaction between *D*. *suzukii* and *Z*. *indianus* showed that to some extent, the performance of each species might depend on the substrate where they developed [43]. However, a structured study to analyze the competition between *D*. *suzukii* and *D*. *melanogaster* showed that one species might have a significantly greater competitive advantage [42]. In that study, the presence of *D*. *melanogaster* significantly reduced the emergence and egg-laying of *D*. *suzukii* [42].

Understanding that an insect’s selection of an oviposition site is a crucial decision with downstream consequences for population dynamics [44], this study evaluated the abundance of drosophilids co-occurring in the field with *D*. *suzukii* in Brazil. The study also assessed the effects of different intra- and interspecific densities, as a proxy for competition, on the oviposition behavior of *D*. *suzukii* females and the interactions between the coexisting competitors *D*. *suzukii* and *Z*. *indianus*.

## Materials and methods

### Fruit sample collections and drosophilid diversity assessment

Strawberry fruits (*Fragaria* x *ananassa*), cultivar ‘Festival’ (GCREC-Dover, 1995) [45] were collected from a strawberry field with seedlings transplanted on March 29, 2020, in Atibaia municipality, São Paulo (23°04’16”S, 46°40’52”W). Strawberry plants were cultivated in open beds 40 m long, in a total area of 0.2 ha. Plants were drip-irrigated every two days. The fruits were collected twice: in October (130 fruits) and December (150 fruits) of 2020. Overripe fruits were collected from the ground and brought to the laboratory to assess the emergence of drosophilids. The fruit was collected randomly around the strawberry field, at 13 places for the first collection and 15 places in the second collection, with ten fruits collected at each place. Overripe fruits were chosen for field collection because the study’s primary goal was to detect the presence of both species co-occurring in the field. The results indicated that both species can occur simultaneously in the same fruit [46]. The fruits were maintained in the laboratory under controlled conditions (R.H. = 60%, L:D = 12:12, T = 26°C). They were placed in 500-mL plastic pots with a layer of vermiculite to absorb moisture, with ten fruits in each pot. For 16 days, the emergence of drosophilids was evaluated daily, and emerging insects were identified under a stereoscopic microscope, using a dichotomous key [47] and the taxonomic description by Van der Linde [48].

### *Drosophila suzukii* and *Zaprionus indianus* intraspecific and interspecific competition bioassays

#### Insect rearing

Individuals of *D*. *suzukii* were obtained from a laboratory colony of Embrapa Clima Temperado, Pelotas, São Paulo (31°48’13.96"S 52°24’41.40"W). Individuals of *Z*. *indianus* were obtained from guava fruits collected in the fields at the Escola Superior de Agricultura (ESALQ; 22°42’30"S 47°38’30"W). The insects used were kept in the artificial diet for at least three generations prior to the bioassay, under controlled conditions (R.H. = 60%, L:D = 12:12, T = 26°C). The artificial diet used for insect rearing and the bioassay followed the artificial diet suggested by Andreazza (2016) [49], composed of cornmeal, sugar, brewer’s yeast, agar, propionic acid, and Nipagin^®^. All the laboratory colonies were kept and the bioassays were conducted in the laboratory in controlled conditions (R.H. = 60%, L:D = 12:12, T = 26°C). The diet was previously tested in populations of *Z*. *indianus* to assure the same survival conditions as *D*. *suzukii*, preventing factors other than competition from influencing the survival of *Z*. *indianus*.

#### Development bioassay

The effect of different egg densities on the developmental parameters of *D*. *suzukii* and *Z*. *indianus* was assessed under inter- and intraspecific competition. Eight densities (ranging from 8 to 400), each with 5 repetitions, were tested using the artificial diet [49]. Plastic cups with a volume of 50 mL were filled with 15 g of artificial diet. After 24 h the diets were inoculated by manually transferring eggs of *D*. *suzukii* and *Z*. *indianus* for the interspecific competition, or eggs of only *D*. *suzukii* or only *Z*. *indianus* for the intraspecific competition, to form the densities: 0.55, 0.69, 1.38, 2.76, 4.14, 6.89, 13.79, or 27.59 eggs/g of diet (i.e., 8, 10, 20, 40, 60, 100, 200, or 400 eggs per cup). The percentages of egg-pupa and pupa-adult viability were measured by counting the pupae and adults, respectively. The mean development time was calculated as the number of days from egg to adult, and the sex ratio was assessed based on counts of all adults 48 h after emergence.

#### Fecundity bioassay

To assess the effects of inter- and intraspecific competition on female fecundity, all adults that emerged from the development experiment were separated into larger plastic cages (380 mL), with one cage for each repetition at each density and allowed them to reach maturity for twenty-four hours for *D*. *suzukii* and four days for *Z*. *indianus*. These intervals were based on our laboratory observations in preliminary tests with the different densities where the female flies showed oviposition-probing behavior as reported elsewhere [9, 50, 51]. In the case of *D*. *suzukii*, such behavior was considered as an initial potential gateway for its competitor *Z*. *indianus*. After this maturation period, a 50-mL plastic cup with 15 g of the artificial diet free of eggs was added to the cage, and adults were allowed to oviposit on the diet for 24 h. Then the diet cup was replaced with a new one and the oviposited eggs were counted under a stereoscopic microscope. This process was repeated for six consecutive days, a period during which we observed a decrease in the eggs laying by the females of *D*. *suzukii*, the primary pest in the substrate (S1 Table). Fecundity was estimated based on the number of eggs per female in each treatment.

#### Leslie matrix

The Leslie matrix was used to describe the population growth, taking into account the stages [52] of *D*. *suzukii* and *Z*. *indianus*. The biological parameters in the matrix were survival and fecundity, which describe changes in population size based on the changes in their values [53, 54]. The model can be represented by the equation:

$$X_{t+1}=Ax_{t}$$

where *X* determines the population size at time *t+1* as a function of time *t* and *A* represents the n × n matrix:

$$\text{A}=\left[\begin{array}{cccc} F1 & F2 & F3 & F4\\ S1 & 0 & 0 & 0\\ 0 & S2 & 0 & 0\\ 0 & 0 & S3 & 0 \end{array}\right]$$

The first line in the matrix indicates “F” values, determining the fecundity of the individuals in each age stage, and the diagonal with “S” values indicates the survival of individuals between life stages [52].

In the equation, *x*_*t*_ represents the stage of the individual present at time *t*, with population growth. The Leslie matrix A is the equation term governing the population growth. The initial values for each age or stage are given by the column matrix, written as:

$$x_{t=}\left[\begin{array}{c} a\\ b\\ c \end{array}\right]$$

Each row of the column matrix represents the initial density of an insect life stage. In the present study, only the first row was filled, with the initial egg density.

Based on the evaluation of data from the development and fecundity bioassay employing the Leslie Matrix with 10 time-step projections, three densities were selected of the eight densities tested, to represent a low (8 eggs), a medium (60 eggs), and a high (400 eggs) density scenario to define the matrix model conditions.

### Behavior bioassay

#### Choice behavior of *Drosophila suzukii* with eggs of *Zaprionus indianus* or with eggs of *D*. *suzukii*

A free-choice assay was used to evaluate the effects of the presence of inter- or intraspecies eggs on the oviposition behavior of *D*. *suzukii* females. In 100-mL plastic cages, combinations of egg-infested diet at different densities versus a non-infested diet (control) were tested. A mean of 0.53 g of diet placed in Eppendorf caps was manually infested with eggs from diets of the laboratory colony of *Z*. *indianus* or *D*. *suzukii*. Four egg densities (1, 3, 7, or 15 eggs per diet, i.e., 1.88, 5.66, 13.20, or 28.30 eggs per gram) were used, each with 9 repetitions. Next, two diets were placed in a cage, one free of eggs and the other with eggs of one of the species at the density tested. Five females and three males of *D*. *suzukii* three days old were released into the cage and left in contact with the diets for 24 h under laboratory conditions (R.H. = 60 ±5%, L:D = 12:12 h, T = 26±1°C). After this period the diets were removed and the eggs counted under a stereoscopic microscope. Because of the morphological difference between the eggs of each pest, i.e., *D*. *suzukii* eggs have two respiratory filaments while *Z*. *indianus* eggs have four respiratory filaments [55], it was possible to evaluate the interspecific density. The initial density, manually infected for the bioassay, was subtracted from the final count of eggs in the oviposition behavior assays.

### Statistical analysis and Leslie matrix approach

The data for development and fecundity for intra- and interspecific competition and the data for mean development time were submitted to regression with the curve adjusted to best fit, using the curve-fitting procedure of SigmaPlot v. 12.5. Linear, quadratic, inverse first-order polynomial curves, and exponential growth and decay models were tested to determine the level of significance and R^2^ values. Model selection was based on parsimony (i.e., simplest model with highest adjusted R^2^ value), high F-values, and steep (relative) increases in R^2^ with model complexity. The data for fecundity and development of 3 densities (8, 60, and 400 eggs) were also submitted to a Leslie matrix with a projection of 10 time steps. The data from the two-choice bioassay of intra- and interspecific behavior were analyzed by Student’s *t*-test. The Leslie matrix analysis was performed using R software, and the other analyses were performed using SigmaPlot v. 12.5 (Systat Software, San José, CA, USA).

## Results

### Field collections

**Fig 1:**
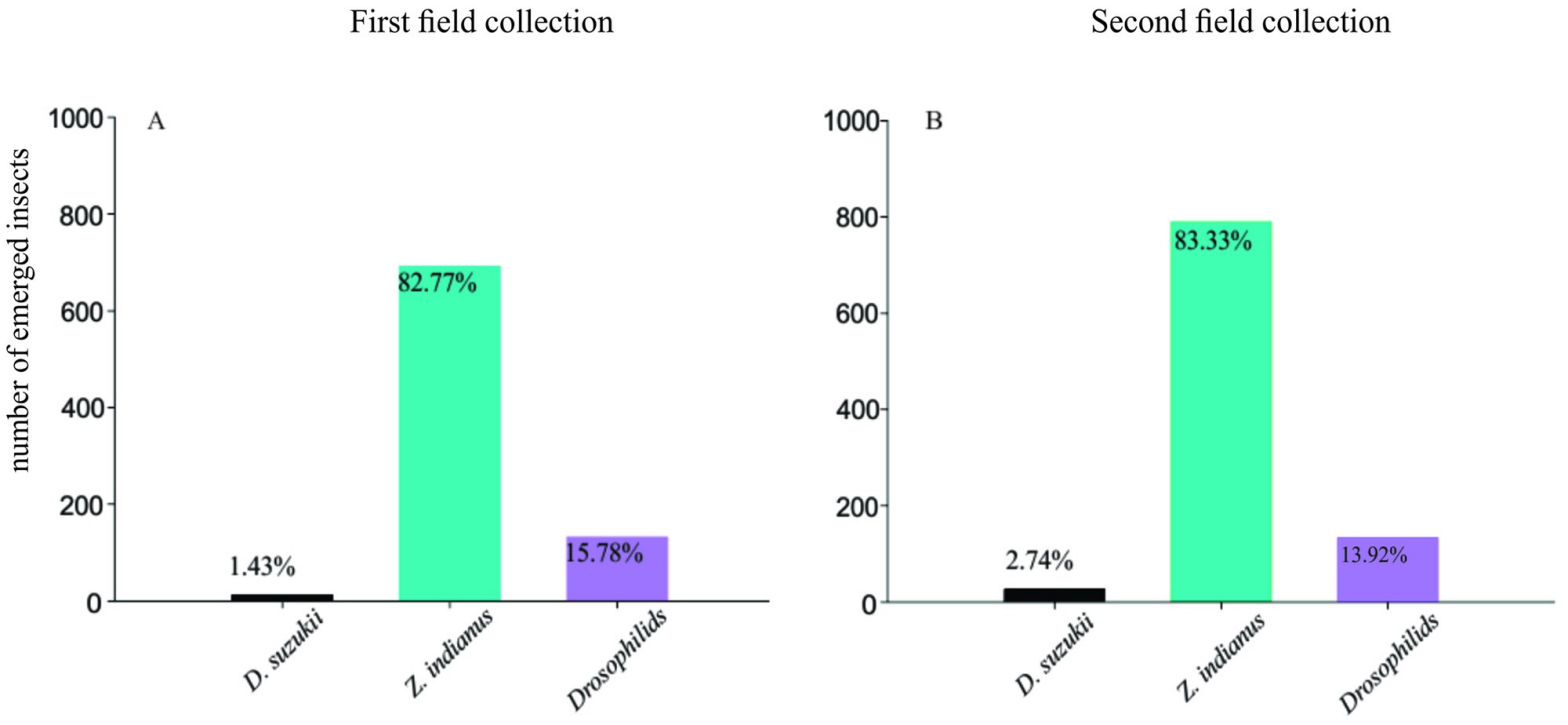
Fruit sample collections and drosophilid diversity assessment. Number of insects emerged from strawberry fruits collected from the field in Atibaia municipality, São Paulo, Brazil (23°04’16”S, 46°40’52”W) in October (A) and December (B) 2020.

*Zaprionus indianus* generally outnumbered the other species. For the first collection date, the total of 836 adults included 12 *D*. *suzukii* (1.43%), 692 *Z*. *indianus* (82.77%), and 132 other *Drosophila* species (15.78%). In the second collection, 948 adults emerged, including 26 *D*. *suzukii* (2.74%), 790 *Z*. *indianus* (83.33%), and 132 other *Drosophila* species (13.92%) (Fig 1).

### Intraspecific and interspecific competition

#### Development bioassay

**Fig 2:**
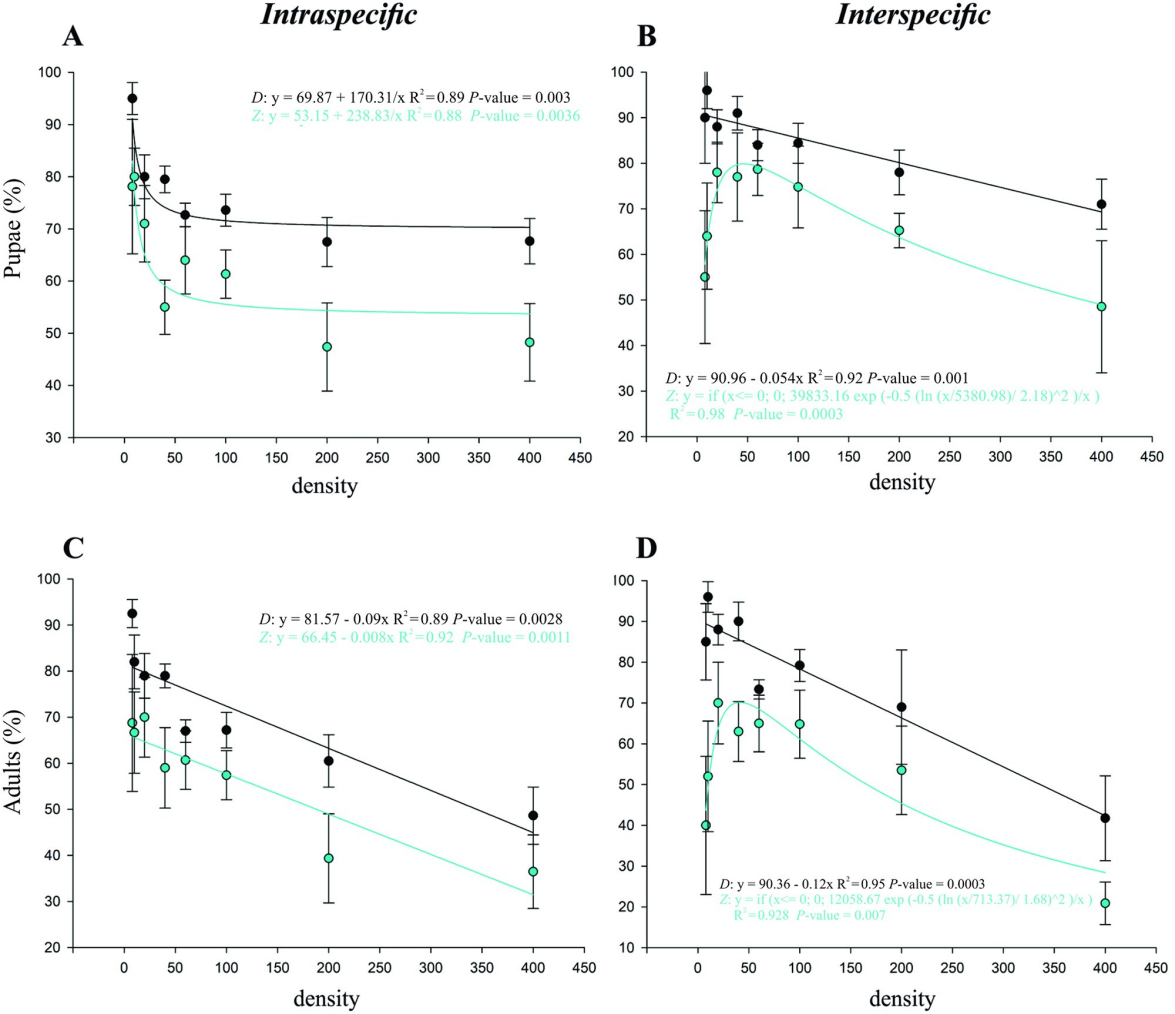
Development bioassay. Percentages of egg-pupa and pupa-adult viability of *Drosophila suzukii* (D) and *Zaprionus indianus* (Z) fitted to regression functions at eight intra- and interspecific egg densities, ranging from 8 to 400. (A) Egg-pupa intraspecific survival. (B) Egg-pupa interspecific survival. (C) Intraspecific emergence percentage of adults. (D) Interspecific emergence of adults.

A linear-regression analysis showed that the effect of density depended on the competing species as well as on the type of competition, intra- or interspecific (Fig 2).

Under intraspecific competition, a density-dependent decrease in egg-pupa survival was observed for *D*. *suzukii* and *Z*. *indianus* (Fig 2A). The data for both species, adjusted to the inverse first-order polynomial curve, showed that an increase in egg density resulted in an initial reduction of pupa survival at lower densities, followed by stabilization of pupa survival at higher densities (Fig 2A, Table 1).

**Table 1:** Summary of regression analyses for percentages of egg-pupa and pupa-adult viability and adult emergence of *Drosophila suzukii* and *Zaprionus indianus* (shown in Fig 2).

	Density	Model	Treatment	Estimated parameters			df_error_	F	p	R^2^
				a	b	y_0_ or x_0_				
*% of pupae*	*intraspecific*	Y = y_0_+(a/x)	*D*. *suzukii*	170.31(83.29–257.32)	-	69.87 (64.62–75.12)		22.93	0.003	0.89
		Y = y_0_+(a/x)	*Z*. *indianus*	238.63(112.25–365.4)	-	53.15 (45.51–60.80)		21.31	0.004	0.88
	*interspecific*	Y = y_0_+a*x	*D*. *suzukii*	–0.05(–0.07– –0.03)	-	90.96 (87.34–94.58)	7	36.06	0.001	0.92
		Y = a*exp(–0.5*(ln(x/x_0_)/b)^2^)/x)	*Z*. *indianus*	39833.1(17402.6–62263.6)	2.18(1.91–2.45)	5380.9 (–917.07–11679.03)	7	62.68	0.0003	0.98
*% emergence*	*intraspecific*	Y = y_0_+a*x	*D*. *suzukii*	–0.09(–0.13– –0.04)	-	81.57(74.03–89.11)	7	23.85	0.003	0.89
		Y = y_0_+a*x	*Z*. *indianus*	–0.08(–0.12– –0.05)	-	66.45(60.44–72.45)	7	34.24	0.001	0.92
	*interspecific*	Y = y_0_+a*x	*D*. *suzukii*	–0.12(–0.15– –0.08)	-	90.36 (84.03–96.68)	7	58.09	0.0003	0.95
		Y = a*exp(–0.5*(ln(x/x_0_)/b)^2^)/x)	*Z*. *indianus*	12058.6(3044.9–21072.3)	1.68(1.21–2.15)	713.37 (–418.1–1844.9)	7	15.7	0.007	0.92

For both species, the percentage of adult emergence showed a decreasing density-dependent pattern, with a noticeable change at high densities (Fig 2C). Adult emergence followed a polynomial linear trend (Fig 2C, Table 1), decreasing continuously even at densities of 13.79 and 27.59 eggs/g of diet (200 and 400 eggs per diet).

With interspecific competition, the patterns of egg-pupa survival and adult emergence changed (Fig 2B and 2D). While *D*. *suzukii* showed a linear density-dependent decrease in both egg-pupa survival and adult emergence, *Z*. *indianus* showed a bell-shaped curve with a peak between density 40 and density 60, indicating that this density range is optimal for the survival of the egg through pupa stages and adult emergence for this species (Fig 2B and 2D, Table 1).

For both cases, intraspecific and interspecific competition, and for both development phases, egg-pupa and adult emergence, the curve for *D*. *suzukii* was higher than the curve for *Z*. *indianus*, i.e., with higher values.

#### Fecundity bioassay

**Fig 3:**
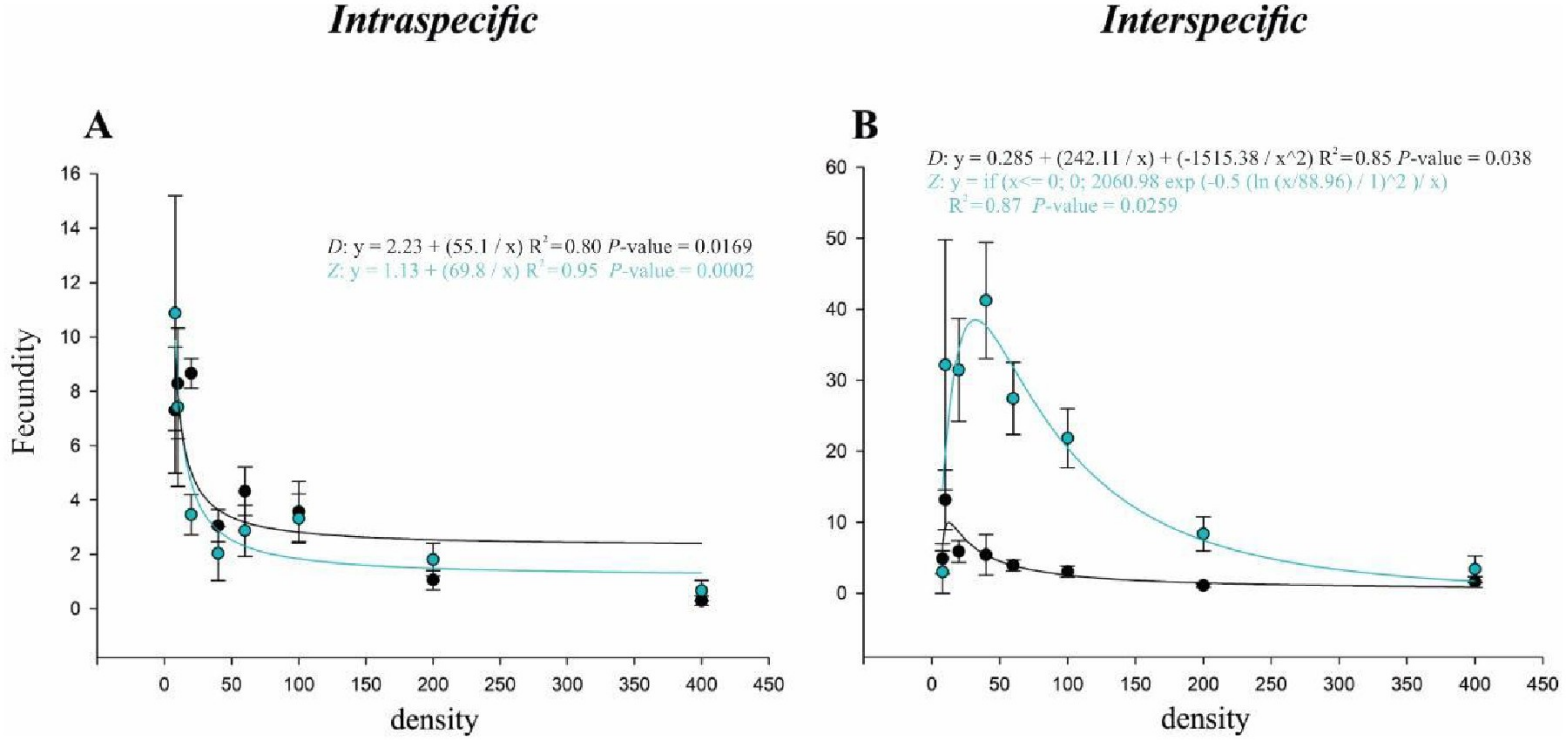
Fecundity bioassay. Fecundity response of *Drosophila suzukii* (D) and *Zaprionus indianus* (Z) fitted to regression functions to eight intra- and interspecific egg densities, i.e., 8 to 400 eggs. (A) Intraspecific mean fecundity per female. (B) Interspecific mean fecundity per female. Black points and curves represent *D*. *suzukii*; green points and curves represent *Z*. *indianus*.

In the intraspecific competition, the data fitted to the inverse first-order polynomial model indicated that the density increase leads to a decrease in female fecundity in each species (Fig 3A, Table 2). However, in the interspecific competition and independently of the species, the fit of fecundity data to the peak-type curves indicated a maximum fecundity per female of approximately 40 eggs for *Z*. *indianus* and 10 eggs for *D*. *suzukii*. Notably, the fecundity per female of *Z*. *indianus* was higher than that of *D*. *suzukii* for all densities tested (Fig 3B, Table 2).

**Table 2:** Summary of regression analyses for fecundity of *Drosophila suzukii* and *Zaprionus indianus* (shown in Fig 3).

Variable	Model	Treatment	Estimated parameters			df_error_	F	p	R^2^
			a	b	y_0_ or x_0_				
*Intraspecific fecundity*	Y = y_0_+(a/x)	*D*. *suzukii*	55.1 (13.97–96.22)	-	2.23 (–0.21–4.74)	7	10.74	0.016	0.80
	Y = y_0_+(a/x)	*Z*. *indianus*	69.8 (48.9–90.7)	-	1.13 (–0.12–2.4)	7	66.85	0.0002	0.95
*Interspecific fecundity*	Y = y_0_+(a/x) + (b/x^2^)	*D*. *suzukii*	242.1 (21.25–462.98)	–1515.3 (–3256.5–225.74)	0.28 (–3.65–4.22)	7	6.71	0.0383	0.85
	Y = a*exp (–0.5*(ln(x/x_0_)/b)^2^)/x)	*Z*. *indianus*	2060.9 (828.63–3293.3)	1 (0.51–1.5)	88.96 (–11.62–189.55)	7	8.27	0.02	0.87

#### Mean development time

**Fig 4:**
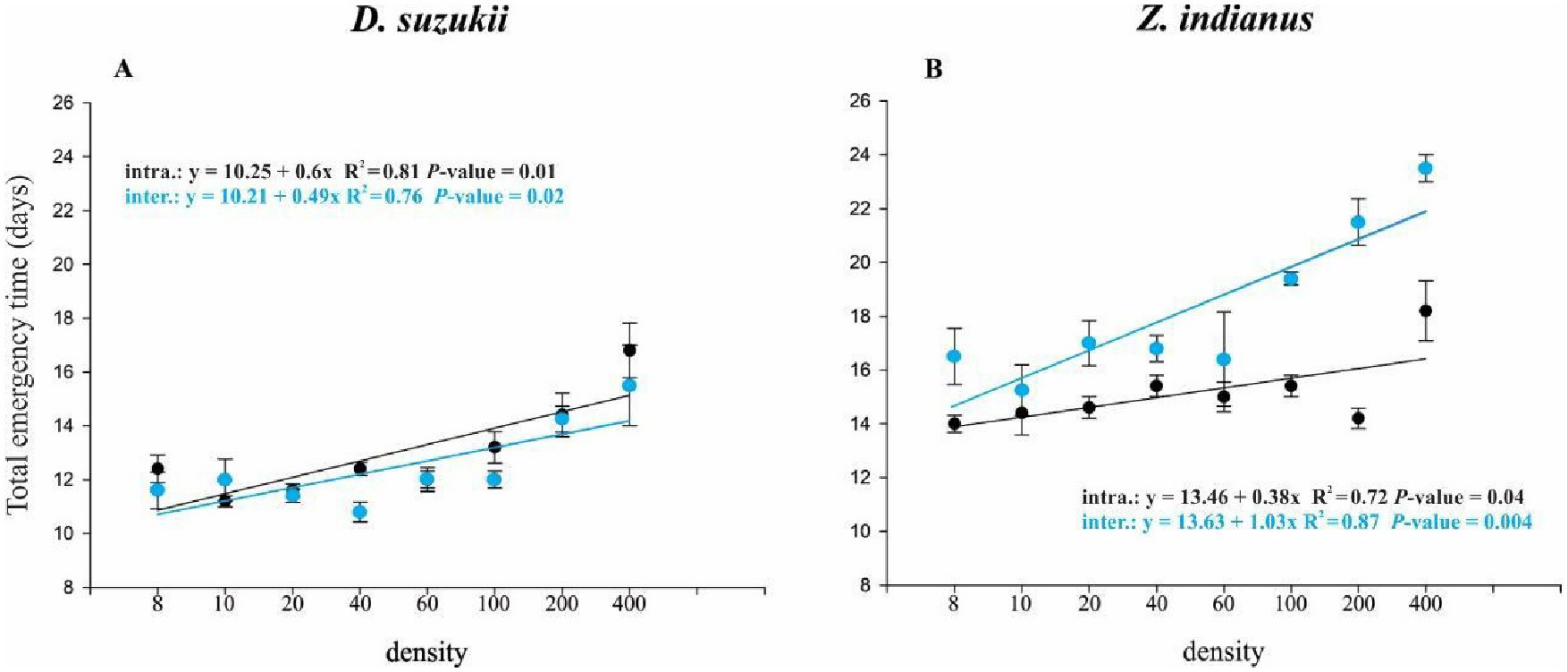
Development time. Mean emergence times of *Drosophila suzukii* (D) and *Zaprionus indianus* (Z) fitted to regression functions in the 8 different densities. The black line and points represent intraspecific competition; the blue line and points represent interspecific competition.

The intraspecific and interspecific competition trajectories for *D*. *suzukii* were significantly fitted to a linear regression (Table 3). The development time increased with the increase in density, with a mean difference of 4 days between the highest and lowest densities. With respect to the type of interaction, intra- or interspecific, even though the intraspecific trajectory was higher than the interspecific line, the development times overlapped (Fig 4A, Table 3). Similarly to *D*. *suzukii*, the egg-adult development time for *Z*. *indianus* increased with the increase in density, with a mean difference of 4 days and 6.88 days from the lowest and the highest density tested for intra- and interspecific competition, respectively (Table 3). The mean development time of *Z*. *indianus* showed a regressive inversion pattern compared to the regressions of *D*. *suzukii*, where now the mean development time of *Z*. *indianus* was longer under interspecific competition than under intraspecific competition (Fig 4B, Table 3).

**Table 3:** Summary of regression analyses for mean emergence times of *Drosophila suzukii* and *Zaprionus indianus* (shown in Fig 4).

Variable	Model	Treatment	Estimated parameters		df_error_	F	p	R^2^
			a	y_0_ or x_0_				
*D*. *suzukii*	Y = y_0_+a*x	*intraspecific*	0.6 (0.18–1.03)	10.25 (8.08–12.42)	7	12.07	0.01	0.81
	Y = y_0_+a*x	*interspecific*	0.49 (0.07–0.91)	10.21(8.09–12.33)	7	8.3	0.02	0.76
*Z*. *indianus*	Y = y_0_+a*x	*intraspecific*	0.38 (0.01–0.75)	13.46 (11.61–15.21)	7	6.61	0.04	0.72
	Y = y_0_+a*x	*interspecific*	1.03 (0.47–1.60)	13.63 (10.77–16.48)	7	20.08	0.004	0.87

#### Leslie matrix

**Fig 5:**
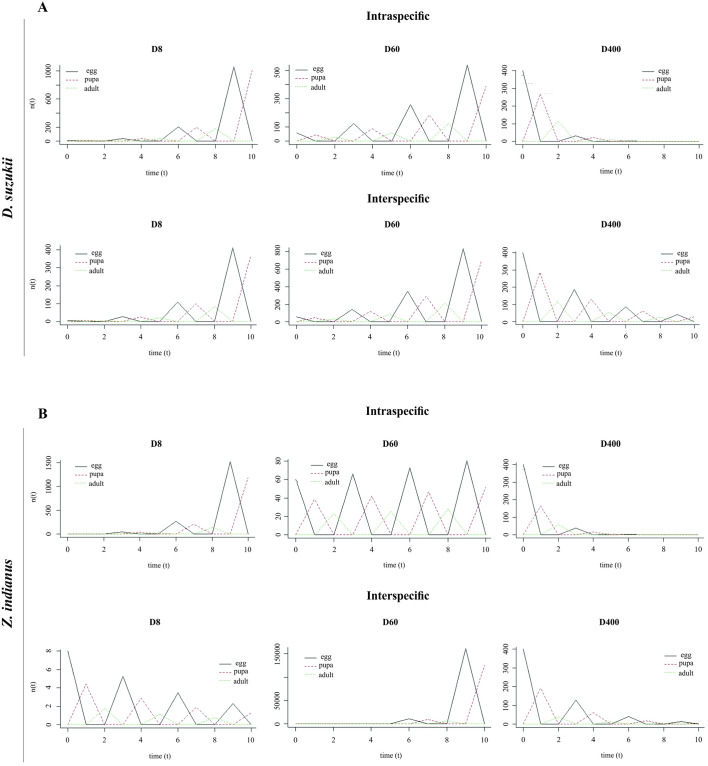
Leslie matrix simulations showing population sizes for life stages of *Drosophila suzukii* (A) and *Zaprionus indianus* (B) from intra and interspecific combinations in scenarios of low (D8), moderate (D60), and high (D400) densities.

For *D*. *suzukii* under intraspecific competition, the characteristics of the trajectory indicated a growth trend of the population as a projection over time for the low-density scenario (Fig 5A). The same growth pattern was apparent in the trajectory under medium density, but with a reduction to half of the final population size. Under high density, the population tended to decrease with time (Fig 5A). A similar pattern was apparent in interspecific competition, with a reduction of the population size compared with the intraspecific competition (Fig 5A).

For *Z*. *indianus* under intraspecific competition and in the low-density situation, the pattern of the trajectory was similar to *D*. *suzukii* (Fig 5B). Under medium density, cyclic oscillations of population growth were seen for all stages of *Z*. *indianus* (Fig 5B). Under high density, as was observed for *D*. *suzukii*, the population growth declined over time. In the interspecific competition, a different pattern was apparent for low density, where, as for high density, the population growth declined (Fig 5B).

### Behavior bioassay

**Fig 6:**
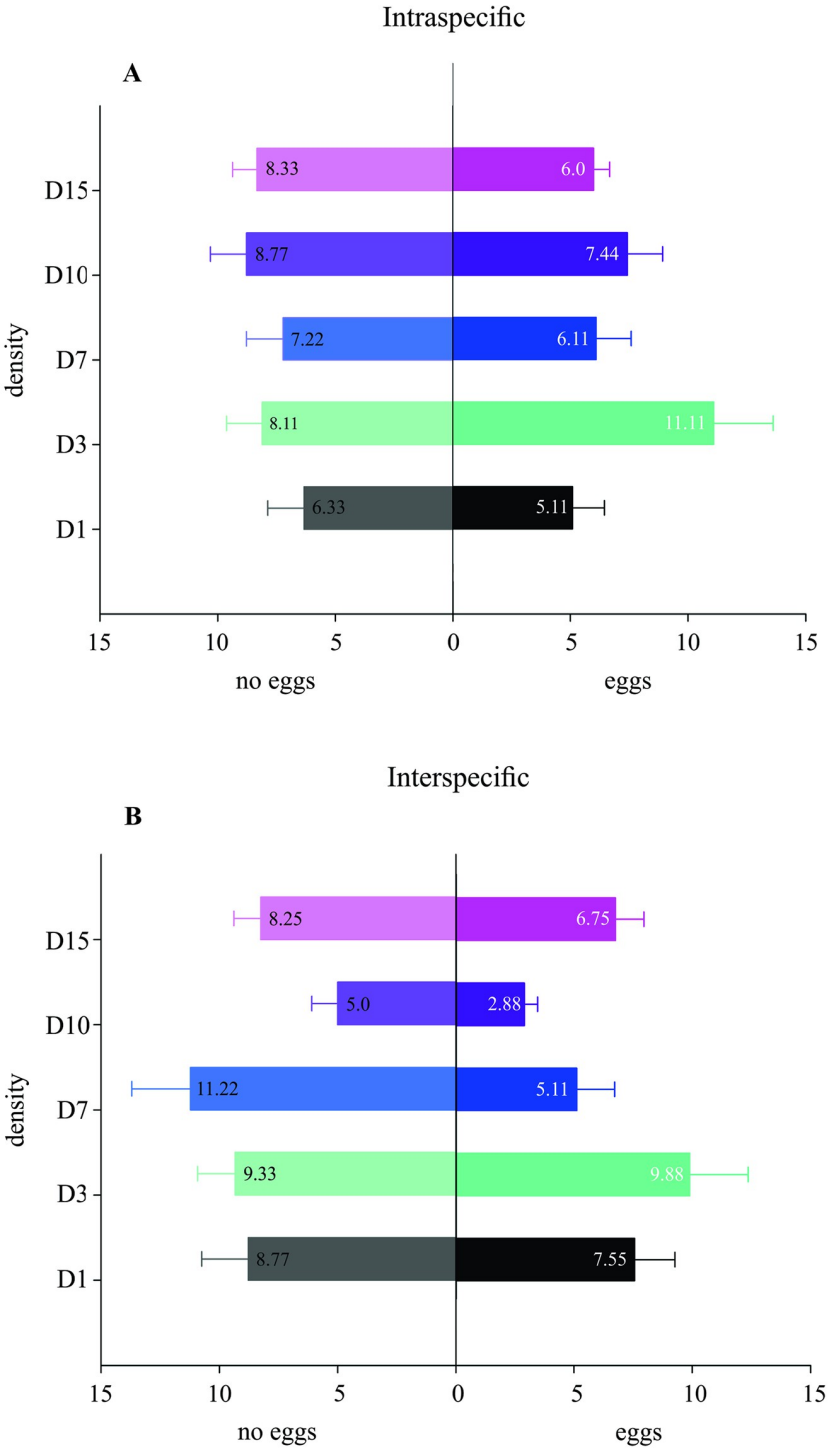
Choice behavior of *Drosophila suzukii* with eggs of *Zaprionus indianus* or with eggs of *D*. *suzukii*. Experiments indicating the total eggs laid by *D*. *suzukii* on a diet previously infested with different egg densities (1, 3, 7, 10, or 15 eggs) versus a blank diet. (A) Intraspecific: oviposition behavior of *D*. *suzukii* in one diet with eggs of *D*. *suzukii* versus a blank diet (no eggs). (B) Interspecific: oviposition behavior of *D*. *suzukii* in one diet with eggs of *Zaprionus indianus* versus a blank diet (no eggs).

In the two-choice bioassay, independently of the density, no significant differences were found in the number of eggs laid by *D*. *suzukii* females allowed to oviposit on either egg-infested or control artificial diet (*P*-value > 0.05, Fig 6A). Similarly, *D*. *suzukii* showed no preference between the control diet and the diet that was previously infested with eggs of *Z*. *indianus* at any of the densities tested (*P*-value > 0.05, Fig 6B). The mean number of eggs laid was between 6.33 ±1.54 (density 1) and 8.77 ±1.54 (density 10) for the control diet, and between 5.11 ±1.35 (density 1) and 11.11 ± 2.53 (density 3) for diets that were previously infested with *D*. *suzukii* eggs.

Similarly, to the intraspecific bioassay, in the bioassay with eggs of *Z*. *indianus*, independently of the density, *D*. *suzukii* showed no preference (*P*-value > 0.05, Fig 6B). The mean number of eggs laid was between 5.0 ±1.09 (density 10) and 11.22 ±2.48 (density 7) for the control diet, and between 2.88 ±0.56 (density 10) and 9.88 ±2.47 (density 3) for diets previously infested with *Z*. *indianus* eggs.

## Discussion

The two drosophilid species *D*. *suzukii* and *Z*. *indianus* occur together in several regions of the world, including Brazil [55–59]. When co-occurring, *Z*. *indianus* is consistently found to significantly outnumber *D*. *suzukii* [43]. Our field collections of strawberry fruits provided substantial evidence concerning the co-occurrence of the two species previously reported, with a higher number of *Z*. *indianus* than *D*. *suzukii* [60, 61]. Nevertheless, a study focusing on field collections with fruits and attractant-baited traps should be encouraged to assess the differences in both species’ population sizes. Under these density conditions, both interspecific and intraspecific competitions are expected to induce changes in important fitness traits of these species. Based on these numerical differences between the two species and knowing that *Z*. *indianus* is an opportunist that requires naturally damaged or previously infested fruits to complete its life cycle [1], we experimentally investigated the roles of competition types (inter- and intra-) in the survival and development dynamics of the two species. Our results from laboratory experiments showed that the biology of *D*. *suzukii* was affected by egg density and partially by the competition type (intra- and interspecific), mainly when observing an inversion of fecundity values, indicated by a higher fecundity of *Z*. *indianus* in interspecific densities. However, the oviposition behavior of *D*. *suzukii* females was not affected by the previous presence of *Z*. *indianus* eggs or by the egg density in the oviposition substrate. On the other hand, *Z*. *indianus* showed higher fecundity in the presence of *D*. *suzukii* eggs. This result suggests that interspecific competition could be more advantageous for *Z*. *indianus* than intraspecific competition.

Our findings showed that independently of the competition type and species, increasing densities had an overall negative effect on development time and fecundity. The emergence time of *Z*. *indianus* was clearly influenced by the type of competition, increasing in interspecific cultures; in contrast, *D*. *suzukii* emerged after similar time periods in both intra- and interspecific cultures. The population density of conspecifics is known to affect individual oviposition rates, impacting fecundity and offspring fitness under resource scarcity [62]. Takahashi and Kimura [63] evaluated the effect of intraspecific competition on the per-capita egg production of *D*. *suzukii* (fecundity). They found a decrease in egg production per capita with the increase in density. Our study also showed a decrease in fecundity with an increase in density. However, we found a similar reduction of *D*. *suzukii* fecundity whether alone or in competition with *Z*. *indianus*.

The effect of interspecific competition on *D*. *suzukii* fitness has been studied mostly with the common fruit fly *Drosophila melanogaster* Meigen as a competitor. In direct interspecific-competition situations, previous oviposition by *D*. *melanogaster* was shown to deter female *D*. *suzukii* from ovipositing in the same substrate [41, 42]. Furthermore, under these interspecific conditions, *D*. *suzukii* larvae showed lower survival than *D*. *melanogaster* larvae [64]. In assays of interspecific competition between *D*. *suzukii* and *Z*. *indianus*, using both an artificial diet and different grape cultivars, the outcome of the competition depended on the oviposition and development substrate [43]. High densities of *Z*. *indianus* did not affect the mortality and development of *D*. *suzukii* when an artificial diet was used, as opposed to grapes, where higher mortality rates were observed [43]. We suppose that the effect of the oviposition substrate would be more marked in the field, since the experiment was conducted with an artificial diet containing a standard quantity of nutrients, differently from field conditions with a natural fruit-based diet [40, 43].

*Zaprionus indianus* responded to the interspecific-density effect with a hump-shaped relationship around densities corresponding to 1.33 and 2.66 eggs/g of diet, indicating a positive density-dependence effect. In this study, the artificial diet was employed to avoid the influence of unknown factors associated with any imbalances in the chemical content of fruit capable of influencing the competitive performance. With the standardized diet, we believe that we could more precisely analyze the effect of competition on the development, fecundity, and behavior of the pests. When showing a competition effect occurring with as many controlled conditions as possible, we presume that the effect found would be accentuated in field conditions.

It is also noteworthy highlighting that in our fecundity experiment; we used individuals, less than one day old, resulting from the previous experiment with different densities (ranging from 8 to 400) that were observed during a 6 days oviposition period. Such experimental conditions could explain some divergences from previous literature [65–68] regarding the oviposition behavior of the studied drosophilids. In fact, biotic and abiotic experimental conditions have been reported to differentially affect the development, behavior, phenology, and reproductive biology of *D*. *suzukii* [69].

In the present study, the results from the projections of the Leslie matrix, although produced through computer simulations, provide evidence for a hypothesis capable of explaining the ecological patterns of the oscillations observed. Allee or hydra effects could be interesting issues for investigation, to explain how low abundance, or as in this study, density dependence causing mortality, would subsequently benefit a population by increasing its equilibrium, especially in cycling populations [70, 71]. Here, the population oscillation patterns observed from the projections were practically the same when analyzing simulations of intra- and interspecific population dynamics for *D*. *suzukii*. That is, increasing oscillations at the densities of 8 and 60 and decreasing oscillations at the density of 400 were observed. However, the oscillation pattern of *Z*. *indianus* in response to intra- and interspecific competition differed significantly from the pattern of *D*. *suzukii*, except at the density of 400. At the intraspecific density of 8, the oscillation pattern was similar to *D*. *suzukii*, but in interspecific simulations, the *Z*. *indianus* population showed a decreasing and oscillating trend. At the density of 60, the intraspecific oscillation showed a slight, gradual increase in the range of oscillation, but under interspecific competition conditions, the oscillation pattern was reversed.

Another critical aspect of these results is the population peaks of these flies in intra- and interspecific simulations. In intraspecific densities of *D*. *suzukii*, the peak numbers of eggs, pupae, and adults decreased with increasing densities, showing a density-dependence effect for all life stages. However, in interspecific densities of *D*. *suzukii*, the peak numbers of eggs, pupae, and adults increased from a density of 8 to 60, and decreased only at the density of 400. The peaks of *Z*. *indianus* decreased dramatically in intraspecific simulations from 8 to 60, and slightly from 60 to 400. However, in interspecific densities, the *Z*. *indianus* peaks increased strongly from 8 to 60, with a significant decrease from 60 to 400. Therefore, intraspecific densities clearly exert stronger negative density-dependent effects than interspecific densities for both species. This result suggests than the co-occurrence between *D*. *suzukii* and *Z*. *indianus* could be beneficial for the persistence of both species. To the best of our knowledge, no studies have combined competitive interactions with the Leslie matrix, as performed here. The Leslie matrix has traditionally been employed to elucidate other aspects of demography, population dynamics, and theoretical ecology, using flour beetles, blowflies, and stinkbugs [30, 72, 73]. Applications of the Leslie matrix to *D*. *suzukii*, *Z*. *indianus*, or other drosophilids have investigated only questions associated with life tables; development, emphasizing temperature; or environmental conditions.

In the two-choice bioassays, the egg density did not appear to affect the oviposition behavior of *D*. *suzukii*, as females showed no preference for diets infested or not with conspecific eggs or *Z*. *indianus* eggs, diverging from a previous report that *D*. *suzukii* females avoided ovipositing in a substrate pre-inoculated with eggs of *D*. *melanogaster* [42]. This difference in behavior could be explained by the ability of some species to mark the oviposition substrate with pheromones. Indeed, males of *D*. *melanogaster* produce the pheromone cis-vaccenyl acetate (cVA) as a marker of the oviposition substrate, which plays a role in different behavioral activities of *Drosophila*, such as male-male aggregation, a reproductive-isolation mechanism; and in modulating oviposition behavior [74, 75]. Although *D*. *suzukii*, *Z*. *indianus*, *D*. *rufa*, and *D*. *auraria* (among others) do not produce this compound [75], they may still be able to recognize it [76], explaining *D*. *suzukii*’s avoidance of substrates previously infected with *D*. *melanogaster* eggs and the lack of this effect with *Z*. *indianus* eggs.

Density dependence has been considered a significant factor influencing biological invasion processes [77]. A high competitive ability has been mentioned as the principal factor responsible for the success of invader species because it allows high rates of population growth [19]. Commonly, competition has been studied by confronting invaders with local species, with results that frequently indicate that the invaders are superior competitors [78, 79]. The present study analyzed two invading species, and therefore both species might maintain the status of the superior competitor. The only difference in terms of advantage would be the time since the invasion event, assuming that the earlier-arriving species would have more time to adjust to its new habitat [80]. Our experimental results provide evidence that competition between *D*. *suzukii* and *Z*. *indianus* limits the numbers of *D*. *suzukii*, suggesting that even between two invader species, larval competition can result in a significant difference in their competitive ability.

In addition to competitive abilities at different intra- and interspecific densities, other factors not investigated in this study can also influence the abundance of drosophilids. Survivorship in drosophilids is associated with temperature. *Zaprionus indianus* withstands high temperatures in regions with wide climate variation, such as Rio Grande do Sul, Brazil [81], although *D*. *suzukii* also shows a degree of tolerance to a tropical climate [82]. Physicochemical characteristics of fruits also seem to affect the establishment and co-occurrence or repellence of both species [50].

In conclusion, our study demonstrated that for *D*. *suzukii*, the presence of *Z*. *indianus* can be considered neutral, since the oviposition behavior of the former did not change in the presence of different densities of *Z*. *indianus*, remaining similar to the behavior in the presence of its own species. The survival and fecundity of *D*. *suzukii* maintained the same pattern in the presence of conspecifics or *Z*. *indianus*, also indicating the lack of effect of *Z*. *indianus* on *D*. *suzukii*. On the other hand, *Z*. *indianus* seems to have produced more offspring in the presence of *D*. *suzukii* than with its conspecifics. These results may explain the high densities of *Z*. *indianus* in the field together with *D*. *suzukii* species. This study highlights the importance of understanding the consequences of the interactions in the field, to anticipate whether these interactions may be transient or permanent. A better understanding of these interactions can help to develop more-appropriate management techniques, anticipating what may occur in the field and thus controlling pests more efficiently.

This study showed how co-occurring fruit flies can use different strategies involving responses in life-history parameters, such as survival and fecundity, when under intra- or interspecific conditions. The responses to these conditions may help to explain the different patterns of coexistence on local and global scales, emphasizing differences among regions and host fruits and providing insights for planning for new crops, especially orchards. Significant evidence supports the idea that competitive interactions can be intensified with climate change, particularly in tropical areas, and new ecological patterns of coexistence and co-occurrence can emerge from this new scenario [83, 84], certainly impacting the distribution of flies in South America and around the world.

## Ethical approval

All applicable international, national, and institutional guidelines for the care and use of animals were considered in the present investigation.

## Informed consent

The authors of this manuscript accept that the paper is submitted for publication in *PLoS One* and state that this paper has not been published or accepted for publication in another journal, nor is it being considered for publication in another journal.

## Supporting information

S1 File Summary of regression analyses for the decrease in the eggs laying by the females of *D*. *suzukii* in intraspecifc and interspecific density. (shown in S1 and S2 Figs).
(PDF)
