# Supplementary material for: Effects of co-occurrence and intra- and interspecific interactions between Drosophila suzukii and Zaprionus indianus
Source: PLoS One. 2023 Mar 30;18(3):e0281806. doi: 10.1371/journal.pone.0281806 (PMC10062649; doi:10.1371/journal.pone.0281806)
Supplement: S1 File — (shown in S1 and S2 Figs). (PDF) [file pone.0281806.s001.pdf]

S1 Table. Summary of regression analyses for the decrease in the eggs laying by the females of *D. suzukii* in intraspecifc and interspecific density. (shown in S Fig 1 and 2)

Variable	Model	Treatment	Estimated parameters			df_{error}	F	p	R^2
			a	b	y_0 or x_0				
Intraspecific fecundity	f = a*exp(-.5*((x-x0)/b)^2)	D8	11.25 (7.81-14.68)	1.04 (0.67-1.41)	3.17 (2.80-3.54)	*3*	28.53	0.01	0.97
		D10	13.38 (5.02-21.73)	0.80 (0.19-1.41)	2.37 (1.81-2.92)	*3*	9.32	0.05	0.92
		D20	34.27 (-4.11-72.66)	0.57 (-0.21-1.36)	2.23 (1.36-3.09)	*3*	2.44	0.23	0.78
		D40	13.30 (9.40-17.20)	1.37 (0.88-1.87)	3.07 (2.60-3.53)	*3*	21.04	0.01	0.96
		D60	19.51 (12.87-26.16)	1.43 (0.82-2.03)	3.88 (3.31-4.44)	*3*	16.47	0.02	0.95
		D100	18.72 (7.01-30.44)	1.24 (0.29-2.19)	2.76 (1.86-3.67)	*3*	3.69	0.15	0.84
		D200	10.37 (8.92-11.82)	0.89 (0.74-1.04)	4.58 (4.44-4.72)	*3*	196.71	0.0007	0.99
		D400	1.70 (1.24-2.16)	1.31 (0.73-1.90)	5.05 (4.54-5.57)	*3*	39.33	0.007	0.98
Interspecific fecundity	f = a*exp(-.5*((x-x0)/b)^2)	D8	3.51 (-1.51-8.55)	4.20 (-16.63-25.04)	3.69 (-4.51-11.90)	*3*	0.08	0.92	0.22
	f = y0+a*x	D10	-0.95 (-1.57- -0.32)	_	9.54 (6.76-12.3)	*5*	15.50	0.01	0.86
		D20	-0.507 (-1.52-0.50)	_	5.82 (1.28-10.36)	*5*	1.64	0.25	0.49
		D40	-1.08 (-2.48-0.31)	_	7.80 (1.67-13.92)	*4*	4.64	0.09	0.73
		D60	-1.92 (-3.13 - -0.71)	_	13.42 (8.01-18.83)	*5*	16.67	0.009	0.87
		D100	-1.97 (-2.86 - -1.07)	_	15.25 (11.26-19.25)	*5*	32.21	0.002	0.93
		D200	-1.92 (-2.56 - -1.29)	_	12.82 (9.97-15.66)	*5*	60.62	0.0006	0.96
		D400	-4.69 (-6.59 - -2.79)	_	31.53 (23.02-40.04)	*5*	40.27	0.0014	0.94

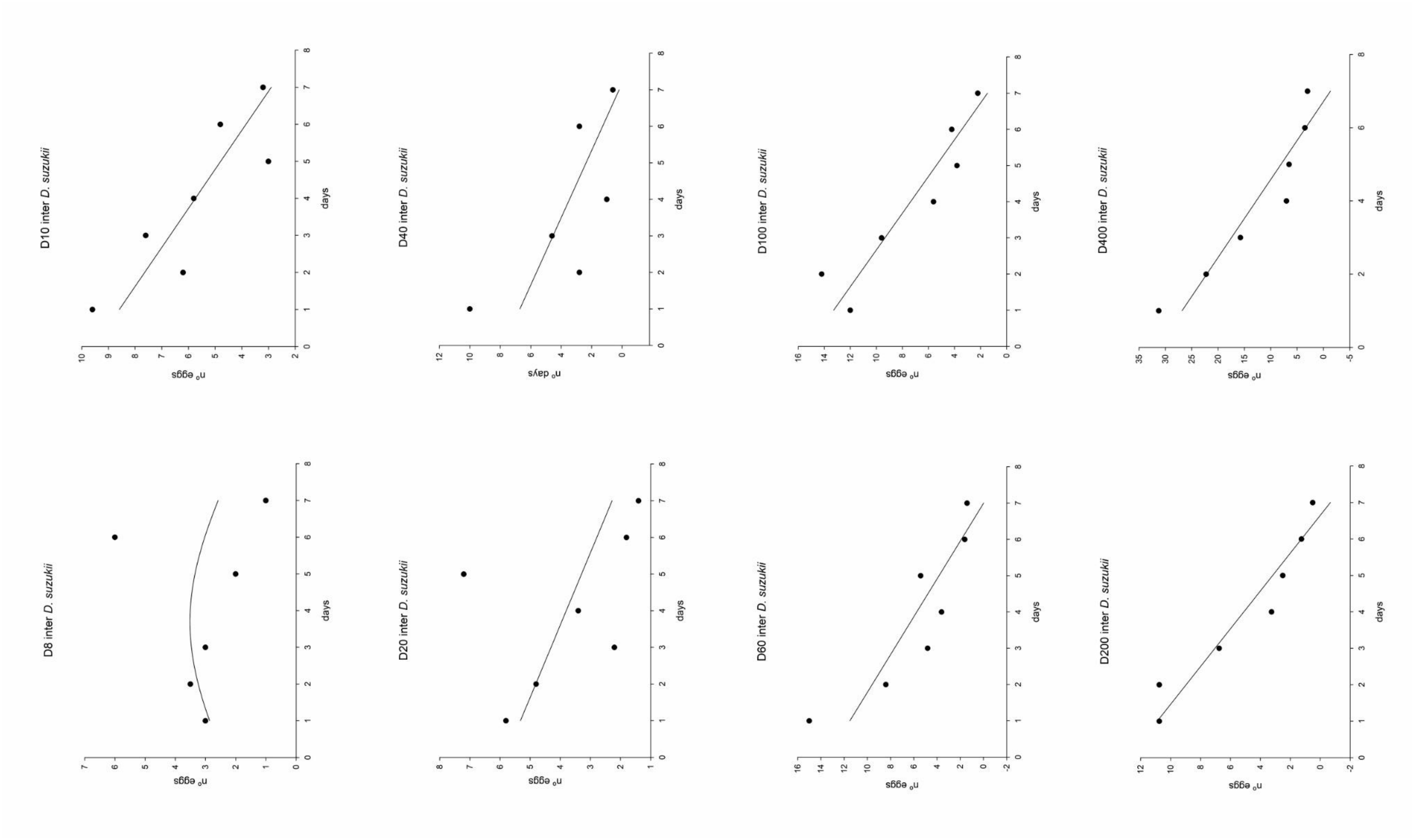

S1 Fig. Peak of fecundity response of *Drosophila suzukii* in interspecific competition during the seven days of evaluation.

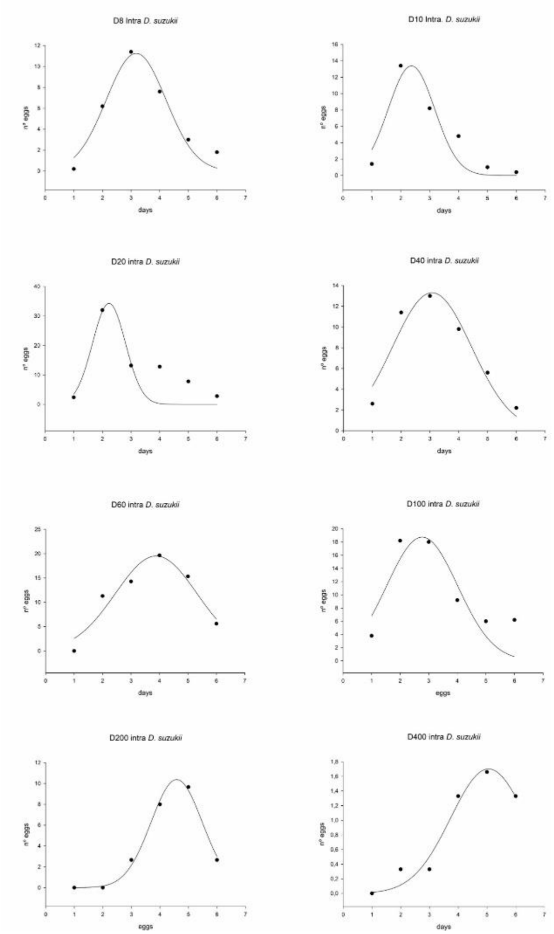

S1 Fig. Peak of fecundity response of *Drosophila suzukii* in **S1 Fig.** **S2 Fig.** Peak of fecundity response of *Drosophila suzukii* in intraspecific competition during the seven days of evaluation.
